# Progress in the discovery of amphipod crustaceans

**DOI:** 10.7717/peerj.5187

**Published:** 2018-07-11

**Authors:** Tri Arfianti, Simon Wilson, Mark John Costello

**Affiliations:** 1 Institute of Marine Science, University of Auckland, Auckland, New Zealand; 2 Research Center for Oceanography, Indonesian Institute of Sciences, Jakarta Utara, Indonesia; 3 Discipline of Statistics, Trinity College Dublin, Dublin, Ireland

**Keywords:** Amphipoda, Crustacea, Taxonomy, Biodiversity

## Abstract

At present, amphipod crustaceans comprise 9,980 species, 1,664 genera, 444 subfamilies, and 221 families. Of these, 1,940 species (almost 20%) have been discovered within the last decade, including 18 fossil records for amphipods, which mostly occurred in Miocene amber and are probably all freshwater species. There have been more authors describing species since the 1950s and fewer species described per author since the 1860s, implying greater taxonomic effort and that it might be harder to find new amphipod species, respectively. There was no evidence of any change in papers per author or publication life-times of taxonomists over time that might have biased apparent effort. Using a nonhomogeneous renewal process model, we predicted that by the year 2100, 5,600 to 6,600 new amphipod species will be discovered. This indicates that about two-thirds of amphipods remain to be discovered which is twice the proportion than for species overall. Amphipods thus rank amongst the least well described taxa. To increase the prospect of discovering new amphipod species, studying undersampled areas and benthic microhabitats are recommended.

## Introduction

Research into the progress of species discovery is of great interest to determine how many species exist on Earth (Costello, 2016). Recent studies on species discovery indicate that about two-thirds of species on Earth have already been described, and show that increasing numbers of people have been describing species new to science (e.g., Appeltans et al., 2012; Costello, May & Stork, 2013; Costello & Chaudhary, 2017). Thus, the present rate of species discovery is being sustained by an increasing number of taxonomists (both professional and amateur), and the relative number of species described per author has been decreasing for decades (e.g., Appeltans et al., 2012; Costello, May & Stork, 2013). These findings indicate that species discovery is flourishing and contradict the view that species discovery and description is facing a crisis (Wheeler, 2004; Wilson, 2004). Regardless of debates about how many species remain to be discovered, there is consensus that the new extinction crisis makes species description urgent to understand life on Earth and use resources sustainably (Costello, May & Stork, 2013; Wheeler et al., 2012).

Coleman (2015) found that the annual rate of species description has increased in recent decades. The most productive taxonomists with numbers of species described, the age distribution of taxonomists, annual rates of new taxa during the careers of four taxonomists, and the most productive taxonomists with working status (having permanent positions or temporary contracts) were also presented. However, trends in subgroups across environments and lifestyles were not studied and these may indicate where new species are most likely to be discovered. In this work, we reviewed trends in species description across all, benthic, pelagic, marine, freshwater, and subterranean amphipods. We used a statistical model to predict the number of amphipod species remaining to be described and analyzed taxonomic effort indicators such as the proportion of “oncer” authors (only ever described one species), the number of single and multiple authorship species, and authors’ publication lifetime and productivity. Although taxonomic effort has been increasing, the proportion of species remaining to be discovered is double that for all species on Earth. Amphipods are thus amongst the least well-known taxa globally.

## Methods

### Data source

Our analysis was based on the world list of amphipod crustaceans in the World Register of Marine Species (WoRMS) (Horton et al., 2016) as recorded on 3rd November 2016. We presented the results of the analysis for all species as well as for marine, freshwater, subterranean, benthic and pelagic species. We classified species as benthic and pelagic based on literature (Barnard, 1969; Bousfield, 1987; Brusca, 1967; Brusca, 1973; Lowry & Myers, 2009; Pillai, 1957; Streets, 1878; Vinogradov, 1988; Vinogradov, 1990). Only species names with accepted status and those that had been checked by the taxonomic editor were analyzed to avoid the over-estimation of the actual number of known species. Species described by Chevreux (1919–1920) were counted as species described in 1919.

### Taxonomic effort

The number of authors is an indicator of taxonomic effort which may influence the rate of description. However, it could be biased by changing authorship practices whereby species are increasingly described by two or more authors. Thus, only the surname of the first author was considered to minimize estimates of effort. Dissimilar names or spelling variants (e.g., Kröyer and Krøyer) for the same author were corrected and counted as the same author. It was assumed that the number of authors with the same surnames at the same time were negligible and random over time (Costello, Wilson & Houlding, 2012). As the changes in description effort reflect the changes in taxonomic citation practices, we also analysed trends in the number of multiple and single authorships.

The trends in taxonomic effort might also be biased if there was a change in the relative productivity of individuals over time. Thus, we counted the proportion of “oncer” authors (described only one species) and calculated Pearson’s skewness coefficient on the number of species named by different authors. Working on another taxon might distract a taxonomist’s attention from describing amphipods. Thus, we analysed the number of non-amphipod species described by the most prolific authors to observe if taxonomists are now more specialized than they were in the 18th century. We also presented linear regressions between the number of non-amphipod species described, the number of amphipods species described, and the first year of an author’s publication. Non-amphipod species description data were gained through the advanced search feature on WoRMS and only non-amphipod species with accepted status were included. As WoRMS is primarily marine species it is possible that authors also described some non-marine species we have not accounted for.

An author’s publication lifetime was calculated as the number of years between their first and most recent species described. If authors’ publication lifetimes have been decreasing over time this may suggest a decrease in the number of amphipod specialists. Accordingly, we provided the linear regression on the publication lifetime against species per year for all authors either including or excluding Dybowsky (who described 97 species in one year).

We counted the number of species described per number of authors in a year. In the early years of discovery, it is easy for authors to find and name new species. After this period, it becomes more difficult and the proportion of species described per number of authors decline. Following Costello, Wilson & Houlding (2012); and Costello, May & Stork (2013), we used Muggeo’s (2003, 2008) method to determine the breakpoint between the increasing and decreasing number of species described per author in the time series.

We analysed whether families and genera which were described first had more species than those newly described by running a simple linear regression analysis between the ages of family against number of species in each family and the age of genus against number of species in each genus. The ratio of species to genera depend strongly on the number of species available and is expected to increase with discoveries overtime. Therefore, we depicted the trend in the number of genera described and the number of species per genus described. The Non-Homogenous Renewal Process (NHRP) was fitted to the description data to make predictions of numbers of amphipod species to be described by 2050 and 2100 by the following equation:

\begin{eqnarray*}\text{Number discovered by year} t= \frac{N}{1+\exp \nolimits (-N\beta (t-\alpha ))} . \end{eqnarray*}

The parameters of the function were: *N*, the total number of species to be discovered; *α*, the year of the maximum rate of discovery; and *β* which describes the overall rate of discovery, with a larger *β* implying a faster rate (Costello & Wilson, 2011; Wilson & Costello, 2005).

## Results

In total, 9,980 accepted names of marine, brackish, freshwater and terrestrial amphipods species were described from 1758 to 2016. There were 221 families, 86 subfamilies, and 1,674 genera. The number of non-accepted species names was 3,332 or 25% of all 13,312 names (Table 1). There were 18 fossil amphipod species discovered up to the year 2016, all of them attributed either to the family Gammaridae or Pontogammaridae, and at least 14 were from the freshwater environment (Table 2). The top-five species-rich genera were *Niphargus, Gammarus, Ampelisca, Caprella,* and *Stygobromus* (Table 3). Six amphipod families had only one species (monotypic) for more than 100 years (Table 4).

**Table 1:** Currently valid and non-valid amphipod families, genera, infraorders, species and subfamilies.

Taxonomy status	Infraorder	Family	Subfamily	Genus	Species
**Accepted**	8	221	86	1,674	9,980
**Non-accepted:**					
-Alternate representation	0	0	2	3	58
-*Interim unpublished*	0	0	0	0	2
-*Nomen dubium*	0	0	0	1	83
-*Nomen nudum*	0	0	0	1	25
-*Taxon inquirendum*	0	0	0	0	6
-Temporary name	0	1	0	5	25
-Unaccepted	3	20	8	210	3,133
**Total non-accepted**	**3**	**21**	**10**	**220**	**3,332**
**Total data**	**11**	**242**	**96**	**1,894**	**13,312**

**Table 2:** Documented fossil occurrences of 18 amphipod species.

Scientific name	Authority	Year of description	Environment	Occurrence
*Palaeogammarus sambiensis*	Zaddach	1864	Freshwater	Eocene-Oligocene Baltic amber
*Gammarus oeningensis*	Heer	1865	Freshwater	Miocene amber
*Hellenis saltatorius*	Petunnikov	1914	Freshwater	Miocene amber
*Gammarus alsaticus*	Van Straelen	1924	Freshwater	Miocene amber
*Praegmelina archangelskii*	Derzhavin	1927	Freshwater	Miocene amber
*Andrussovia bogacevi*	Derzhavin	1927	Freshwater	Miocene amber
*Andrussovia sokolovi*	Derzhavin	1927	Freshwater	Miocene amber
*Praegmelina andrussovi*	Derzhavin	1927	Freshwater	Miocene amber
*Palaeogammarus balticus*	Lucks	1928	Freshwater	Eocene-Oligocene Baltic amber
*Andrussovia vassolevitschi*	Derzhavin	1941	Freshwater	Miocene amber
*Gammarus praecyrius*	Derzhavin	1941	Probably freshwater	Miocene amber
*Gammarus retzi*	Maikovsky	1941	Probably freshwater	Miocene amber
*Palaeogammarus danicus*	Just	1974	Freshwater	Eocene-Oligocene Baltic amber
*Tethorchestia palaeorchestes*	Bousfield & Poinar Jr.	1995	Terrestrial	
*Niphargus groehni*	Coleman & Myers	2001	Freshwater	Eocene-Oligocene Baltic amber
*Palaeogammarus polonicus*	Jażdżewski & Kulicka	2002	Freshwater	Eocene-Oligocene Baltic amber
*Palaeogammarus debroyeri*	Jażdżewski, Grabowski & Kupryjanowicz	2014	Freshwater	Eocene-Oligocene Baltic amber
*Synurella aliciae*	Jażdżewski, Grabowski & Kupryjanowicz	2014	Freshwater	Eocene-Oligocene Baltic amber

**Table 3:** The 35 families with the most species, their number of genera and the year of description of the first and last species as per Horton et al. (2016).

Rank	Family	Number of species	Number of genera	First species described (Year)	Last species described (Year)
1	Lysianassidae	536	82	1830	2015
2	Caprellidae	425	90	1767	2016
3	Gammaridae	414	34	1758	2016
4	Niphargidae	377	10	1836	2016
5	Phoxocephalidae	369	79	1842	2015
6	Maeridae	367	47	1808	2016
7	Talitridae	316	72	1766	2016
8	Ampeliscidae	302	4	1840	2013
9	Stenothoidae	279	45	1815	2015
10	Ischyroceridae	264	43	1808	2014
12	Aoridae	250	25	1843	2016
11	Oedicerotidae	250	46	1842	2014
13	Crangonyctidae	226	7	1840	2016
14	Photidae	225	17	1828	2016
15	Ampithoidae	216	16	1816	2016
16	Leucothoidae	179	5	1789	2015
17	Melitidae	175	26	1804	2016
18	Uristidae	171	24	1774	2014
19	Pontogeneiidae	168	32	1838	2014
21	Corophiidae	158	26	1761	2016
20	Hyalidae	158	12	1830	2016
22	Pleustidae	141	33	1838	2009
23	Dexaminidae	125	12	1813	2016
24	Acanthogammaridae	122	34	1858	2002
25	Liljeborgiidae	119	2	1853	2012
26	Eusiridae	116	10	1780	2015
27	Bogidiellidae	113	37	1933	2016
28	Eulimnogammaridae	112	16	1858	2014
29	Stegocephalidae	109	27	1774	2012
30	Synopiidae	108	17	1853	2013
31	Iphimediidae	105	15	1843	2014
32	Calliopiidae	104	27	1830	2014
33	Hadziidae	91	27	1907	2016
34	Amphilochidae	90	15	1862	2016
35	Podoceridae	89	8	1814	2016

**Table 4:** The age of families consisting of one species (monotypic families).

Family	Age (Year)	Species
Argissidae	147	*Argissa hamatipes* (Norman, 1869)
Baikalogammaridae	142	*Baikalogammarus pullus* (Dybowsky, 1874)
Dairellidae	131	*Dairella californica* (Bovallius, 1885)
Giniphargidae	117	*Giniphargus pulchellus* (Sayce, 1899)
Prolanceolidae	109	*Prolanceola vibiliformis* Woltereck, 1907
Tryphanidae	145	*Tryphana malmii* Boeck, 1871

### Progress in species descriptions

**Figure 1:**
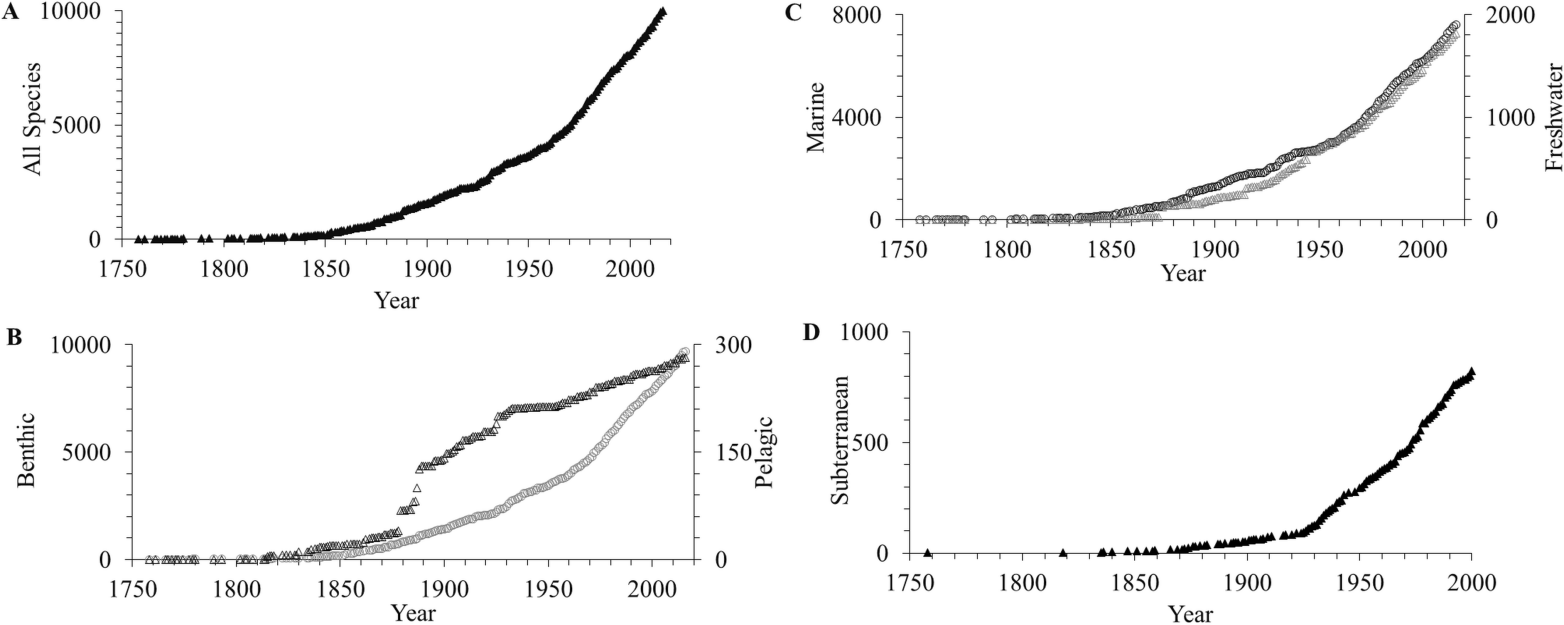
Accumulation curves of amphipod species described per year. (A) all accepted, (B) benthic (gray circles) and pelagic (black triangles), (C) marine (gray circles) and freshwater (black triangles), and (D) subterranean up to the year 2016. Note scales vary.

After an initial period of low discovery for 100 years, descriptions of new species accumulated steadily from the 1850’s to 1950’s, and have since maintained a linear rate of new species descriptions (Fig. 1A). A high proportion of pelagic amphipods were described in the late 19th century, but relatively few have been described compared to benthic species since then (Fig. 1B). Far more marine than freshwater species were described (Fig. 1C). The description of subterranean species was later than for marine and freshwater environments, with most species described since the late 18th century (Fig. 1D).

**Figure 2:**
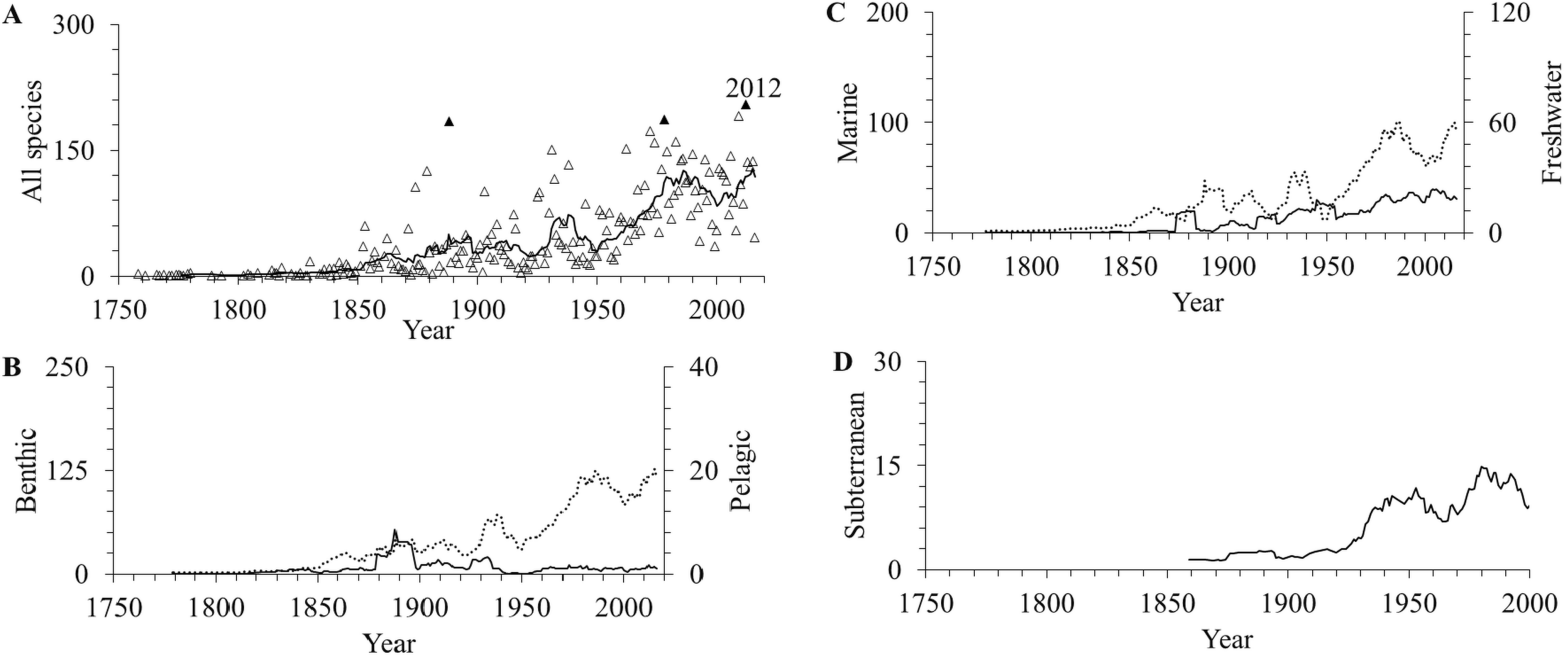
The number of amphipod species described per year. (A) all species, (B) benthic (dotted line) and pelagic (solid line), (C) marine (dotted line) and freshwater (solid line), and (D) subterranean up to the year 2016. Solid triangles indicate top three years for species described. The lines are 10-year moving averages.

The greatest discovery of amphipods was in the 20th century, with the highest peak at 204 species in 2012 (Fig. 2A). A similar pattern also occurred for benthic, marine, freshwater and subterranean species (Figs. 2B–2D). However, the number of pelagic amphipods described had been declining since the 1890s (Fig. 2B). Overall, the average number of species described per year was 48 (95% confidence interval 41–54).

**Figure 3:**
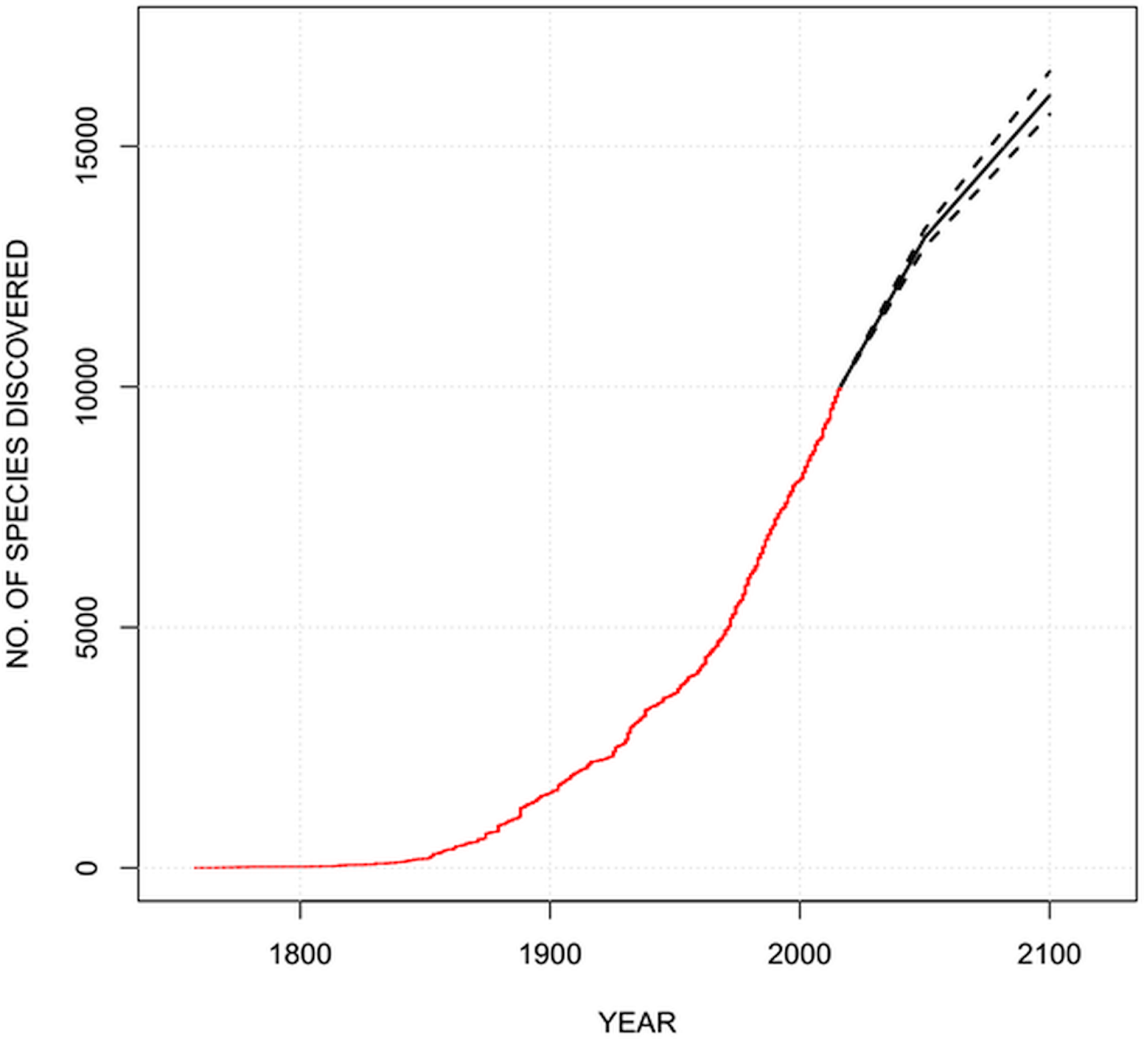
The cumulative number of amphipod species described over time (red line), with the predicted number of species based on the number of species described by 2050 and 2100.

Using the nonhomogeneous renewal process model, with 95% probability, we predicted that by 2050 and 2100, there would be 2,900–3,300 and 5,600–6,600 more amphipod species to be described with the median prediction of 3,100 and 6,100, respectively (Fig. 3).

**Figure 4:**
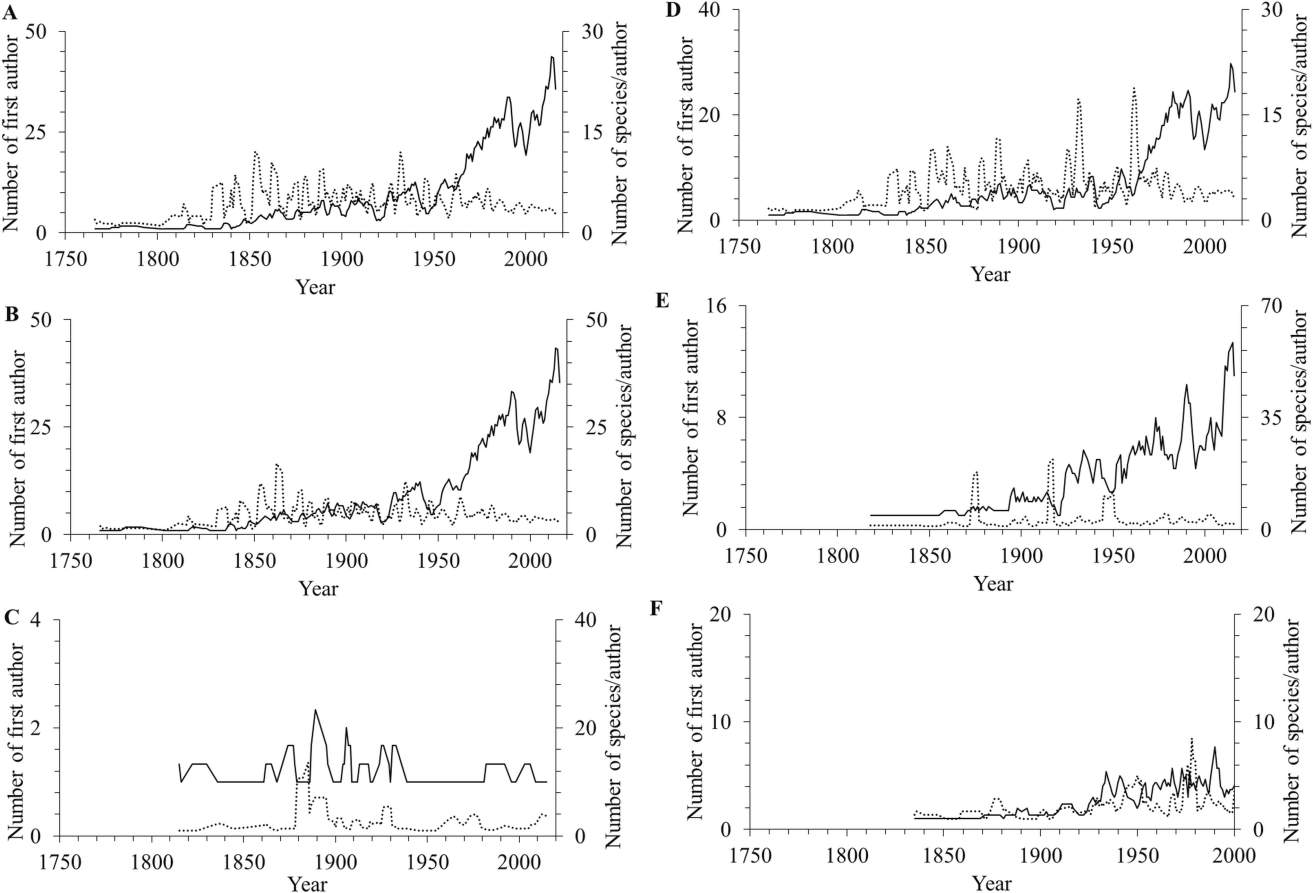
The number of first authors (solid line) and species/author (dotted line) of amphipod species described. (A) all species, (B) benthic, (C) pelagic, (D) marine, (E) freshwater, and (F) subterranean per year up to the year 2016. The lines are three-year moving averages.

**Figure 5:**
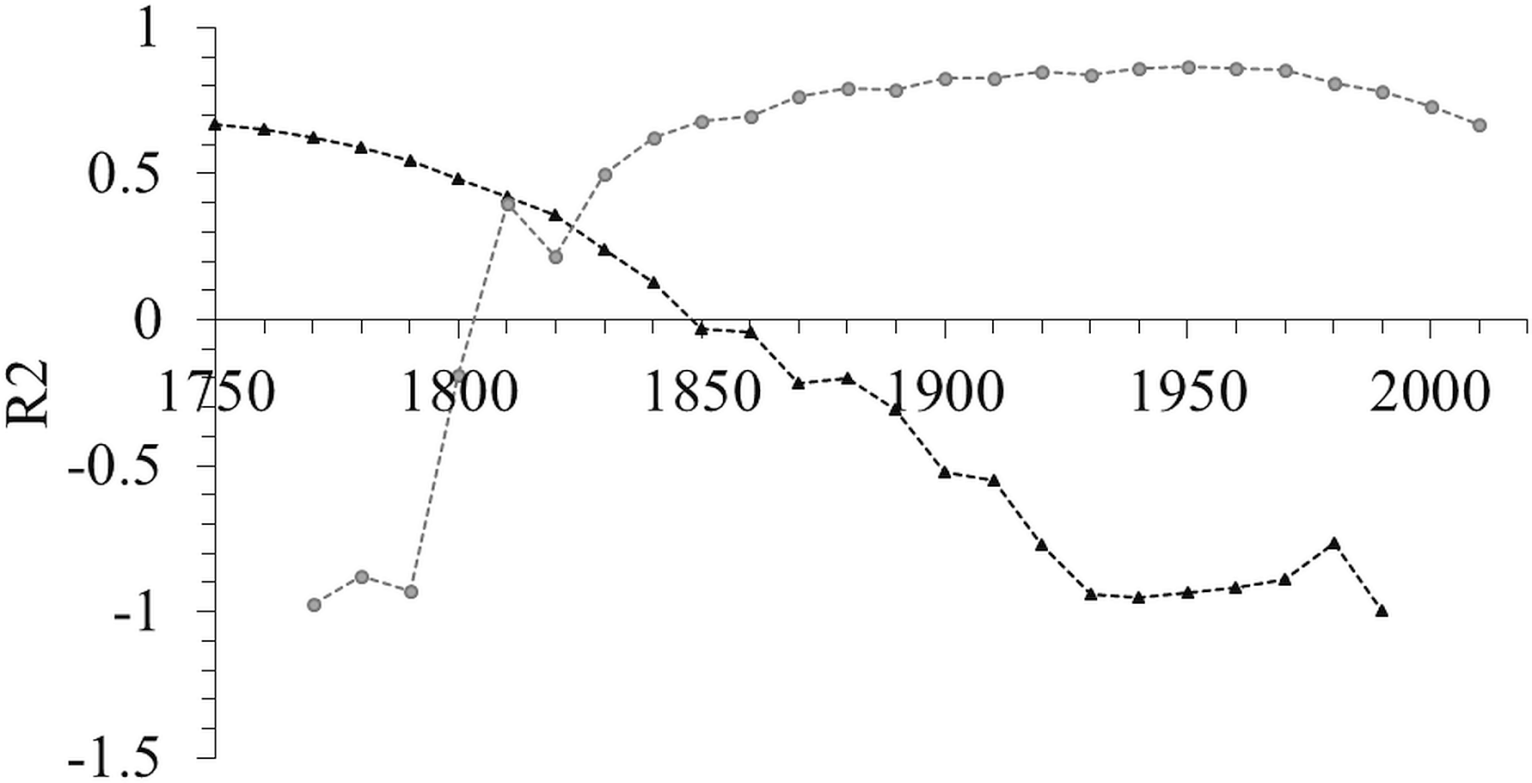
Change in the correlation coefficient of the number of all amphipod species described per author over 25 decades indicating the breakpoint was around 1820. The grey dots indicate the correlation coefficients between the most recent decade and each previous decade. In contrast, the black triangles are the correlation coefficients between the first and each subsequent decade.

The number of authors describing species has increased in recent decades (Fig. 4A). The same increasing pattern was also found in benthic, marine, freshwater and subterranean species (Figs. 4D–4F), while there was no significant change in the number of authors describing pelagic species from the beginning of the 1950s (Fig. 4C). However, despite the increase in the number of authors describing species in recent decades, the number of species described per number of authors has decreased since the 1860s (Fig. 4A). The same downward trend occurred in the benthic, pelagic, marine, freshwater and subterranean species (Figs. 4B–4F). The break point in the time series for all species was in 1820 (Fig. 5). Even though there was a period with high productivity (a high number of species/author) between the 1830s and the 1970s, the overall trend since then has been a decreasing number of species per number of authors.

### Authors as an indicator of taxonomic effort

**Figure 6:**
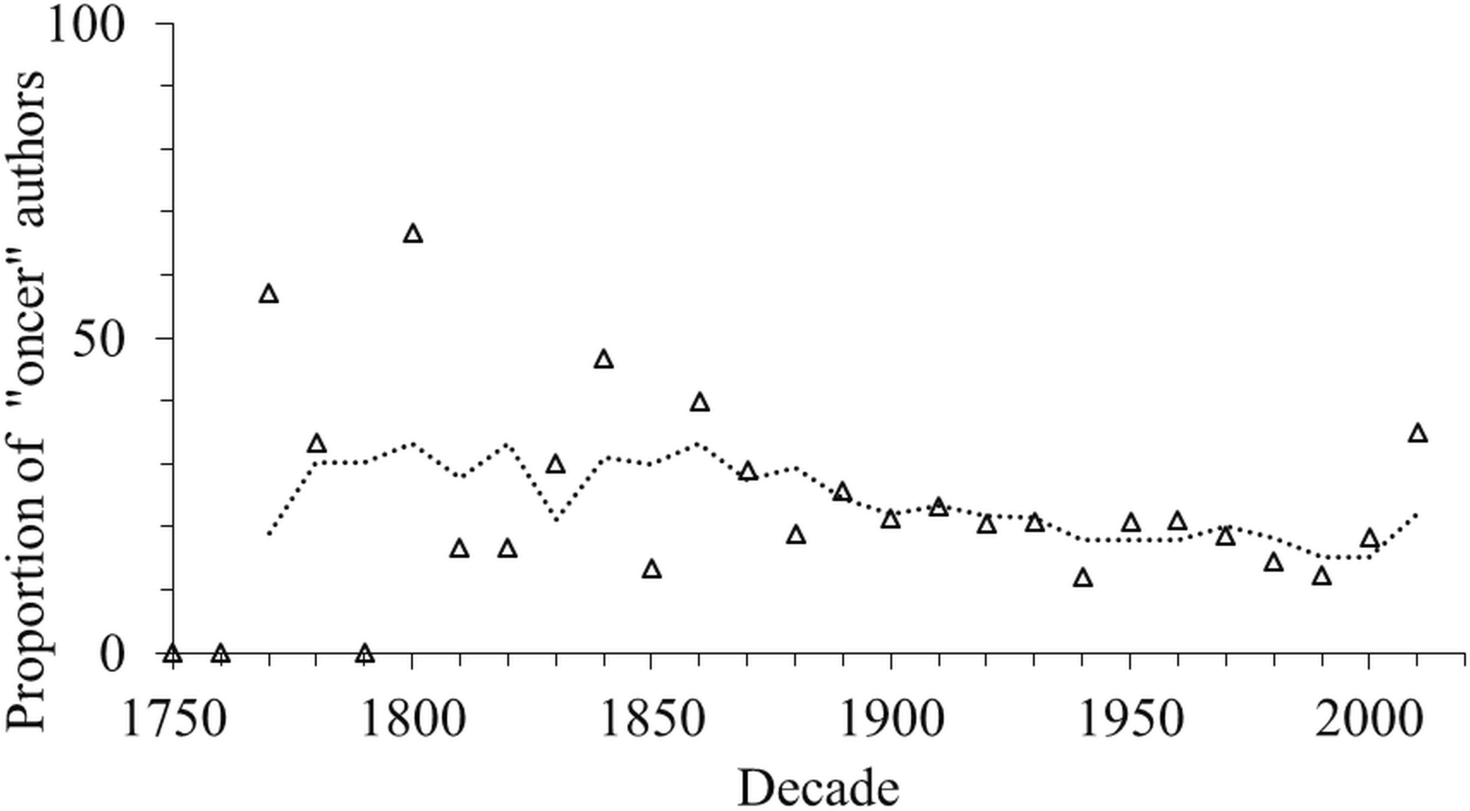
The proportion of authors who described only one species (“oncers”) per decade, up to the year 2016. The lines are three-year moving averages.

The trend in amphipod discovery above might be due to the increasing number of “oncers,” people who only describe one species. However, we found no trend in the proportion of oncers for more than one century and in the recent decade the highest proportion of “oncers” was only 35% (Fig. 6). Also, the skewness values were positive and were around 1.0 with an exception in the 1990s, which had high skewness (Fig. S1). Furthermore, it appears that “non-oncers” authors were responsible for almost all the new species descriptions (98%) since the 1850s, whereas “oncers” authors described only 2% of species.

**Figure 7:**
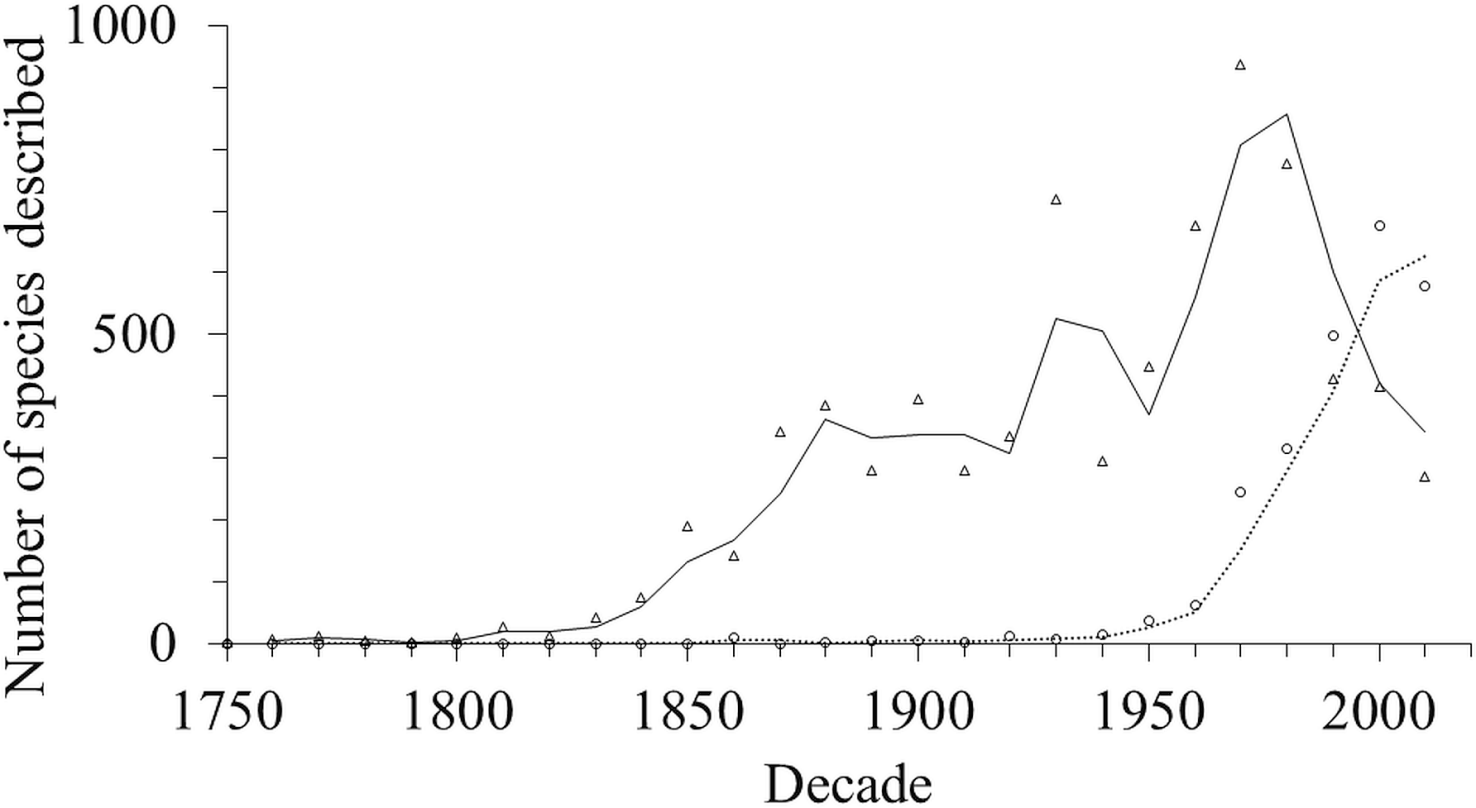
The number of amphipod species described with one (triangle) and multiple (circle) authors.

At the beginning of amphipod species descriptions in the 1750s, single authorship was the norm. Multiple authorship emerged more than 100 years later in the 1860s. Since the 1950’s the number of multiple authorships has been increasing (Fig. 7).

Among the 638 people involved in describing amphipod species from 1758 to 2016, 26 people described more than 90 species which we considered to be the most prolific authors (Table S1). We found that the average publication lifetime of all authors was nine years. However, there was no significant trend (*R*^2^ < 0.1, *P* > 0.05) in authors’ publication lifetimes over years for all authors, whether Dybowsky (who described 97 species in one year) was included or not (Fig. S2). Thus, there is no evidence of any change in author productivity since the 1850’s.

We found a significant trend (*R*^2^ < 0.5, *P* < 0.05) in the number of species between the “old” families or genera and the newly described families or genera (Fig. S3) reflecting a gradual accumulation of species over time through discoveries. Thus, families and genera which were described first had more species than those newly described. As observed in the number of species described, there was also an increase in the number of genera described and species to genus ratios in recent decades (Fig. S4).

There was no significant relationship (*R*^2^ = 0.0003, *P* > 0.05) between the number of amphipod and non-amphipod species described by the most prolific authors (Fig. S5A). However, there was a weak but significant relationship (*R*^2^ = 0.16, *P* < 0.05) in the number of non-amphipod species described with the first year of an author’s publication (Fig. S5B) indicating that recent authors are describing fewer non-amphipods.

## Discussion

### Species description rates

The number of species described for the entire fauna (benthic, marine, freshwater and subterranean species) increased in recent decades and the apparent decrease in the year 2016 may be explained by the time delay in entering new descriptions into WoRMS (Costello, Wilson & Houlding, 2012). As observed in the number of species described, there was also an increase in the number of genera described and species to genus ratios in recent decades. The decreased trend found in the number of pelagic species described for the past century is likely because pelagic species are more geographically widespread than benthic and thus are described earlier (Costello et al., 2015). The peak in the number of pelagic species described in 1879 was due to the contribution of a single author, Claus, who published three new genera and 29 new hyperiid amphipod species (Claus, 1879).

The nonhomogeneous renewal process model predicted that about one third of the described species would be described by 2050, and two-thirds more by 2100. These findings contrast with findings for all species on Earth and all marine species using the same model (Appeltans et al., 2012; Costello, Wilson & Houlding, 2012), and other assessments using proportions of undescribed species (Appeltans et al., 2012) and expert assessments (reviewed by Costello & Chaudhary, 2017). In these other cases, at least half and sometimes 80% of species are considered to have been described, and about two-thirds overall. Thus, amphipods appear to be only half as well described as other taxa.

The period of greatest discovery in the 20th century may have been influenced by the Global Taxonomy Initiative, Census of Marine Life (O’Dor et al., 2012) and PEET Program (Partnerships for Enhancing Expertise in Taxonomy) (Rodman & Cody, 2003). The peak in 2012 was due to the significant contribution of Ren (2012) in the second volume of Fauna Sinica which yielded 39 new species. Another contribution was from Lowry and coworkers who described 21 new species in the family Pachynidae, and established a new family Acidostomatidae and a new subfamily Conicostomatinae (Lowry & Stoddart, 2012; Stoddart & Lowry, 2012). Twenty-four new species of leucothoids from Japan (White & Reimer, 2012a; White & Reimer, 2012b; White & Reimer, 2012c) and six new species of *Rhinoecetes* from the South-eastern Australian Shelf were also contributing factors (Just, 2012). That families and genera which were described earlier had more species than those described later indicates that the major evolutionary lineages of amphipods have already been described. Thus, new species tend to be from already known families and genera.

### Taxonomic effort

The increased number of people involved in describing species indicated increased taxonomic effort. Other studies have similarly reported the increasing trend in taxonomic effort (e.g., Costello, Wilson & Houlding, 2012; Costello, May & Stork, 2013; Costello, Houlding & Wilson, 2014; Costello et al., 2015; Essl et al., 2013; Irfanullah, 2013) which must have contributed to the sustained high number of species described (Appeltans et al., 2012). We found no change in skewness (of papers per author per year) over the past century, and that the percentage of “oncers” had not shown any trend in the past century and the largest proportion of “oncers” was only 35%. This ratio was lower than reported in previous research (42%–44%, Appeltans et al., 2012). Neither was there any significant trend in authors’ publication lifetimes or productivity of the 26 most prolific authors over time. By only using first authors we avoided any bias due to increasing multiple-authorships of species in recent decades. However, such co-authors are often first authors on other species, so our results will underestimate rather than overestimate the number of active authors of new species. Thus, there was no evidence of any change in author productivity that may affect description rates. The same results have also been reported for other taxa (Costello, Wilson & Houlding, 2012; Eschmeyer et al., 2010; Joppa, Roberts & Pimm, 2011; Pimm et al., 2010). Another measure of taxonomic productivity is the number of publications, and this is highly correlated with the number of authors per species (Edie, Smits & Jablonski, 2017).

As taxonomy develops, more time is needed to check synonyms and species relationships, to compare species characteristics with established species, and to produce more comprehensive publications (Frank & Curtis, 1979; Poulin & Presswell, 2016; Sangster & Luksenburg, 2015). However, these factors are likely compensated by the efficiency of the latest technology (Eschmeyer et al., 2010), the availability of reputable online database such as WoRMS to check the status of a species, the online availability of original descriptions from the Biodiversity Heritage Library, and greater ease of communication, transportation and improvement in sampling methods (Costello, Wilson & Houlding, 2012; Costello, May & Stork, 2013). For amphipods particularly, there is an extensive taxonomic character database (DELTA) and new techniques on amphipod descriptions initiated by Coleman (2003), Coleman (2006), Coleman (2009) and Coleman, Lowry & Macfarlane (2010), that provide more efficient ways of digital drawing for publication. We found that working on other taxa did not affect the number of amphipod species described. Also, we found that recent authors are having fewer non-amphipods described; thus there is no evidence of authors being distracted from working on amphipods.

## Conclusions

Our results support previous studies on species description rates in finding increasing numbers of authors, a decreasing rate of species being described per authors, no changes in the relative productivity of authors over the past century, and that pelagic species are relatively better named than benthic. Most future new species are likely to be benthic and endemic, and from already known families and genera. However, we also find that two-thirds of amphipod species remain to be described, double the proportion of most other taxa, whether marine or terrestrial.

##  Supplemental Information

10.7717/peerj.5187/supp-1 Figure S1 The skewness in number of species described by different authors in each decade

10.7717/peerj.5187/supp-2 Figure S2 The linear regression of publication lifetime against species/year (*r*^2^ = 0.0003) The correlation is less when excluding Dybowsky who published 97 species/year is excluded (*r*^2^ = 0.0003), for all authors.

10.7717/peerj.5187/supp-3 Figure S3 The linear regression of (A) number of species in a family against the age of family, and (B) number of species in a genus against the age of genus

10.7717/peerj.5187/supp-4 Figure S4 The number of (A) genera described, and (B) species/genus described, up to the year 2016 The lines are five-year moving averages.

10.7717/peerj.5187/supp-5 Figure S5 The linear regression of non-amphipods described against (A) spp described (*R*^2^ = 0.0003, *P* > 0.05), and (B) the first year of author’s publication (*R*^2^ = 0.16, *P* < 0.05)

10.7717/peerj.5187/supp-6 Table S1 A list of the most prolific authors (i.e., described more than 90 species)

## Additional Information and Declarations

### Data Availability

The following information was supplied regarding data availability: Arfianti, Tri; Wilson, Simon; Costello, Mark (2018): WoRMS_Amphipoda_RawData.xlsx. figshare. Dataset. https://doi.org/10.17608/k6.auckland.6213965.v1.
