# Supplementary figures and images for: Progress in the discovery of amphipod crustaceans
Source: PeerJ. 2018 Jul 11;6:e5187. doi: 10.7717/peerj.5187 (PMC6045924; doi:10.7717/peerj.5187)

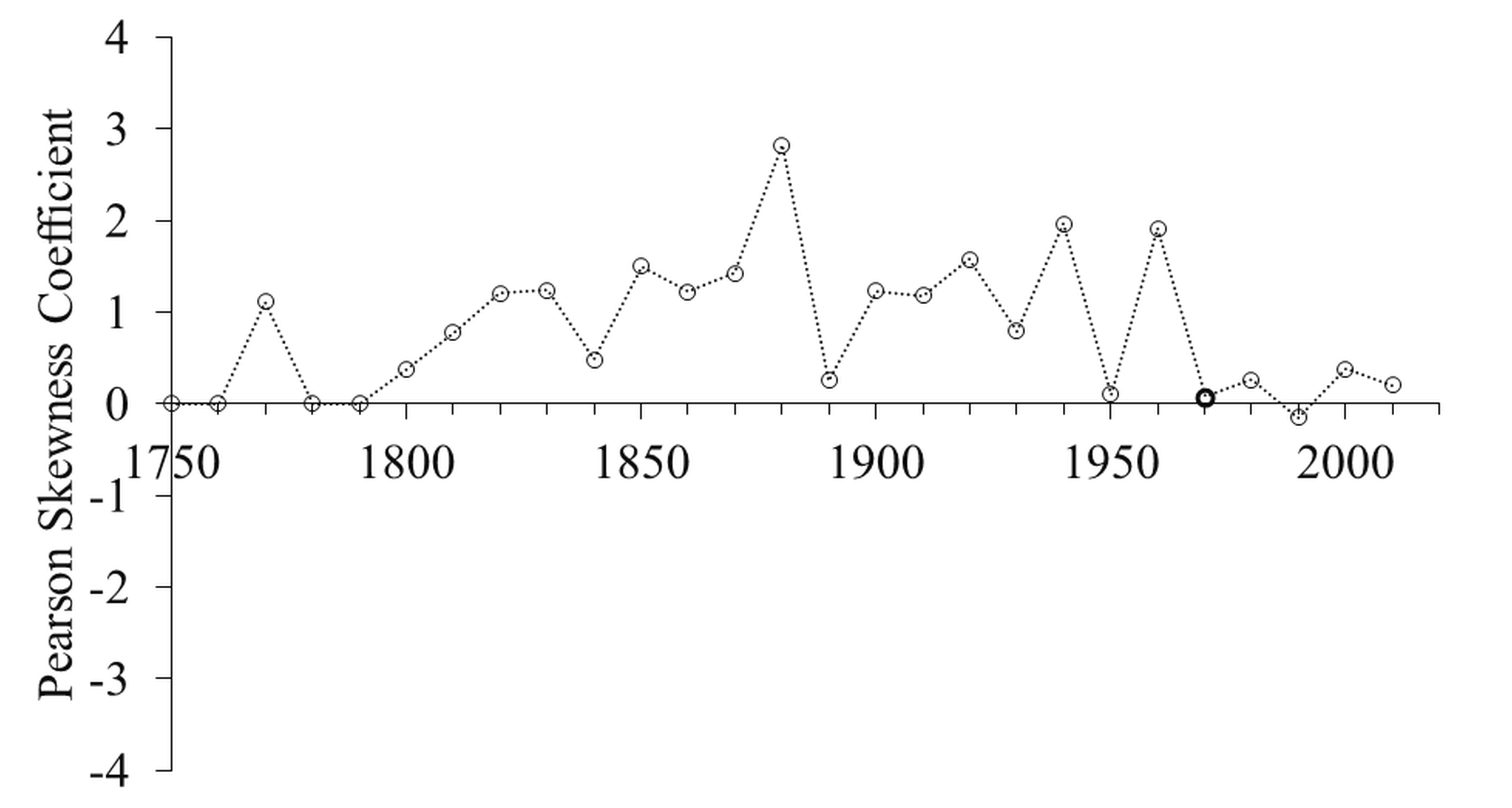

Supplement: Figure S1 [file peerj-06-5187-s001.png]

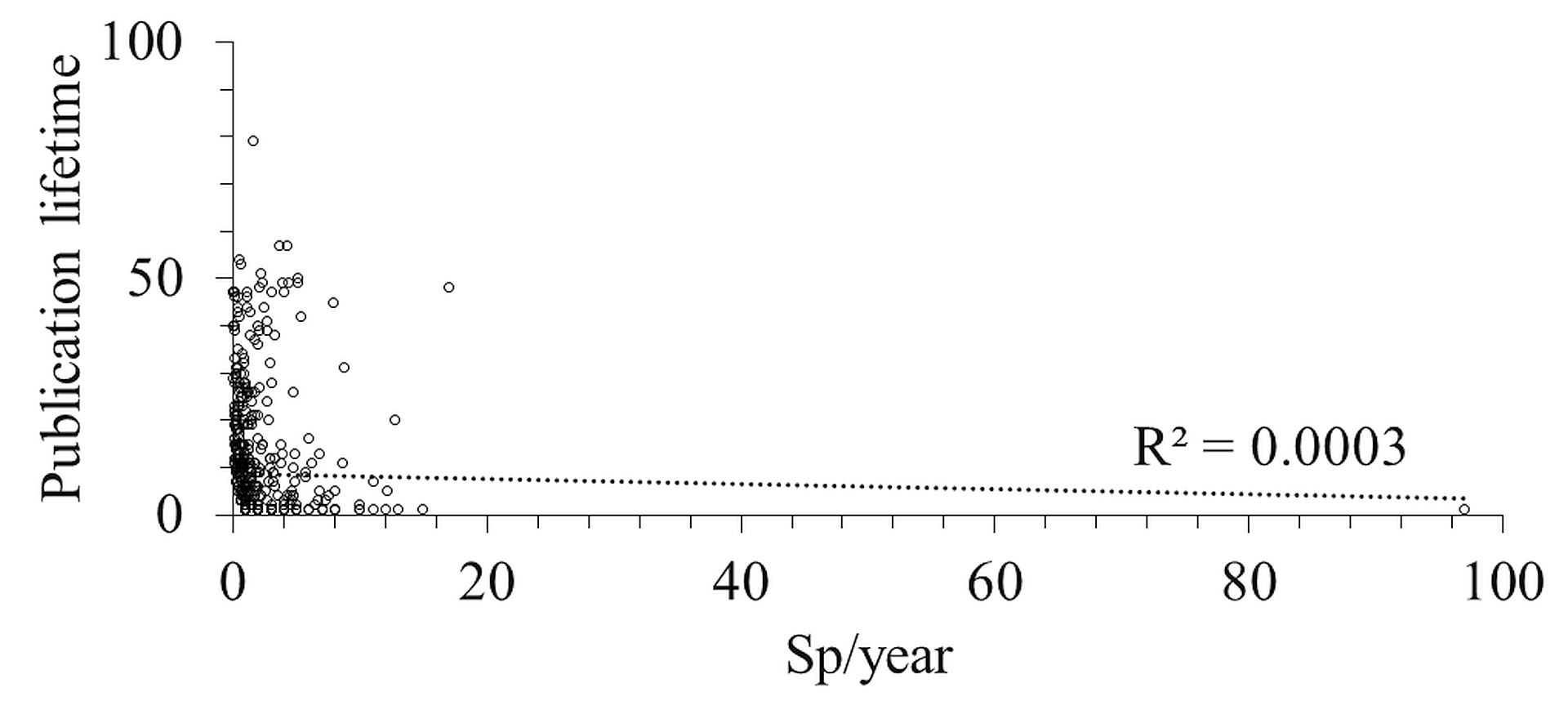

Supplement: Figure S2 — The correlation is less when excluding Dybowsky who published 97 species/year is excluded (r2 = 0.0003), for all authors. [file peerj-06-5187-s002.png]

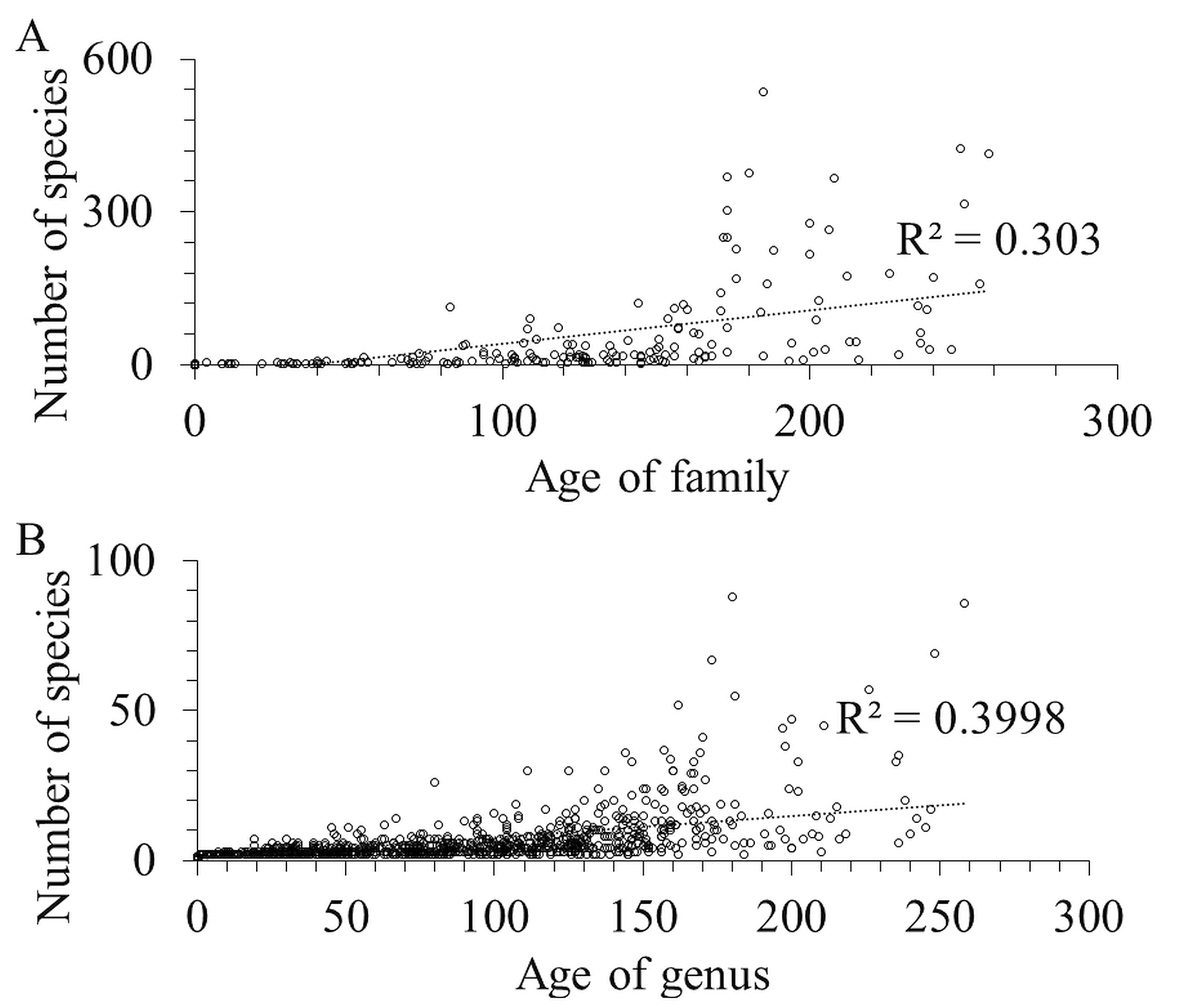

Supplement: Figure S3 [file peerj-06-5187-s003.png]

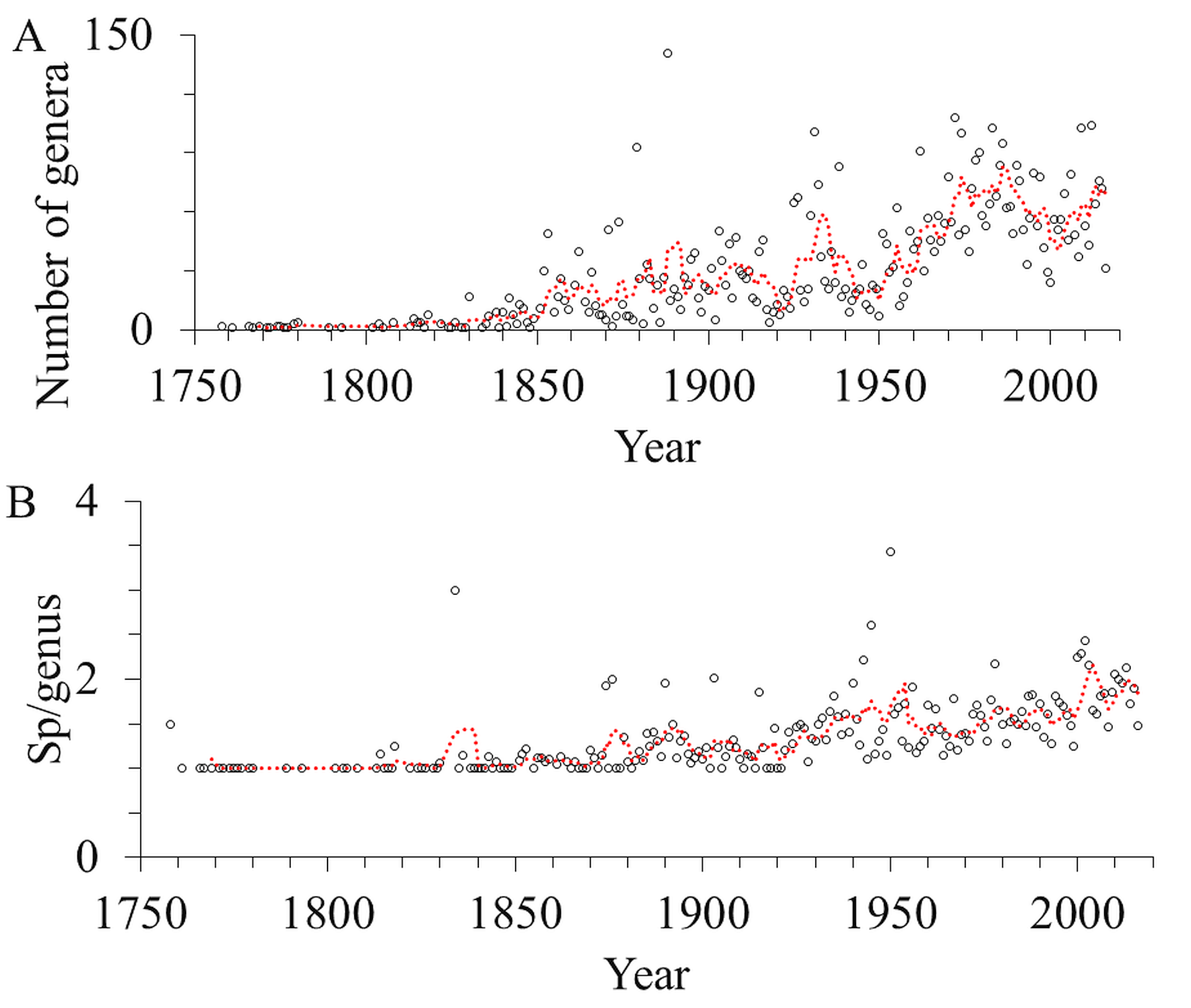

Supplement: Figure S4 — The lines are five-year moving averages. [file peerj-06-5187-s004.png]

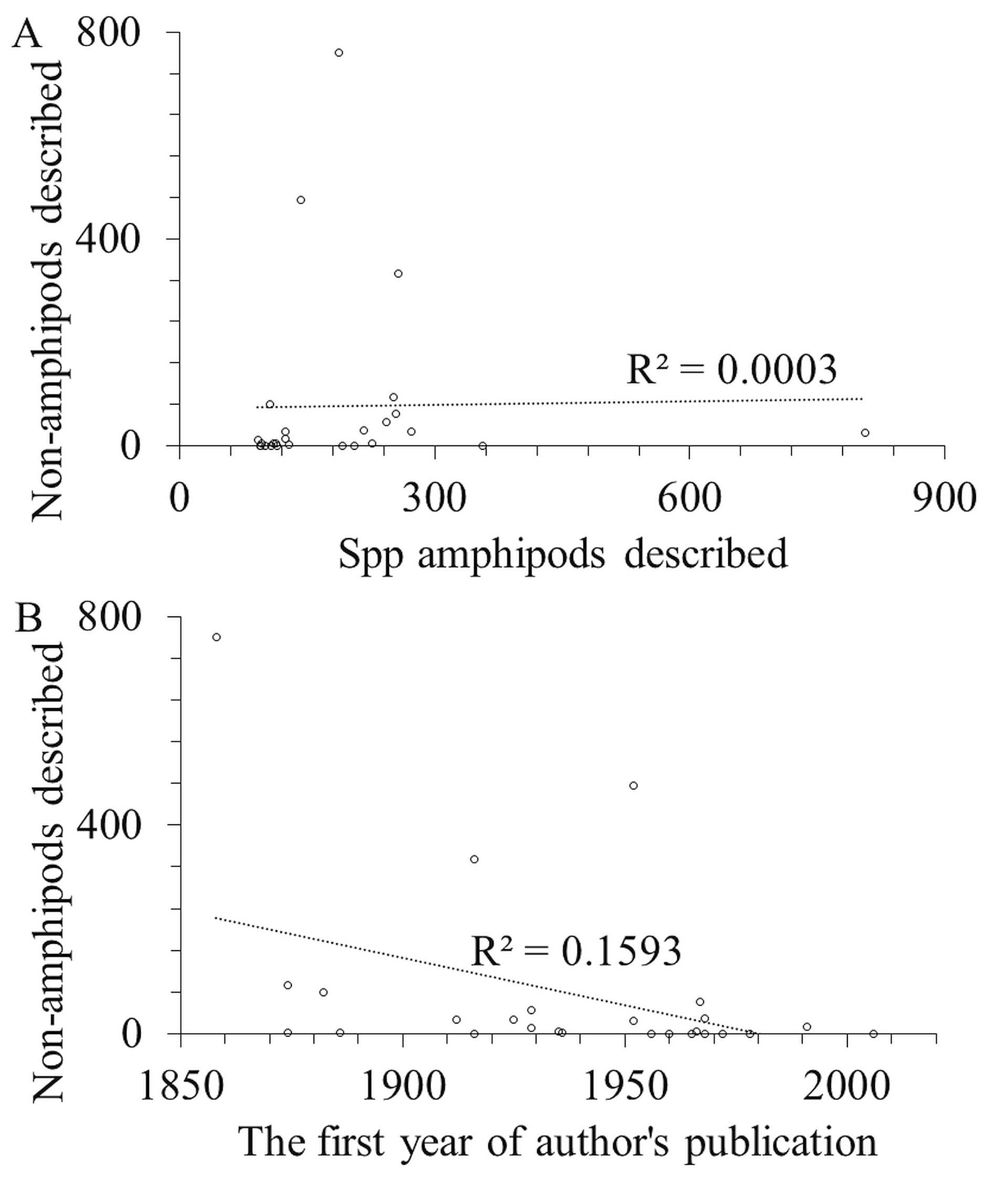

Supplement: Figure S5 [file peerj-06-5187-s005.png]
