# Supplementary material for: Progress in the discovery of amphipod crustaceans
Source: PeerJ. 2018 Jul 11;6:e5187. doi: 10.7717/peerj.5187 (PMC6045924; doi:10.7717/peerj.5187)
Supplement: Table S1 [file peerj-06-5187-s006.docx]

Table S1. A list of the most prolific authors (i.e. described more than 90 species).

| First Author | Species described | | | Publication lifetime (Year) | Species / year | Non-amphipod species described |
| --- | --- | --- | --- | --- | --- | --- |
|  | Total | First | Last |  |  |  |
| J.L. Barnard | 806 | 1952 | 1999 | 48 | 17 | 25 |
| Lowry | 356 | 1972 | 2016 | 45 | 8 | 0 |
| Schellenberg | 272 | 1925 | 1955 | 31 | 9 | 28 |
| K.H. Barnard | 257 | 1916 | 1965 | 50 | 5 | 334 |
| Ledoyer | 254 | 1967 | 1986 | 20 | 13 | 61 |
| Stebbing | 251 | 1874 | 1922 | 49 | 5 | 94 |
| Gurjanova | 243 | 1929 | 1985 | 57 | 4 | 46 |
| Chevreux | 227 | 1886 | 1927 | 42 | 5 | 3 |
| Myers | 217 | 1968 | 2016 | 49 | 4 | 29 |
| G.S. Karaman | 206 | 1960 | 2016 | 57 | 4 | 0 |
| Bousfield | 191 | 1956 | 2004 | 49 | 4 | 0 |
| Sars | 187 | 1858 | 1904 | 47 | 4 | 760 |
| Stock | 143 | 1952 | 1998 | 47 | 3 | 476 |
| Ruffo | 129 | 1936 | 2014 | 79 | 2 | 1 |
| Stephensen | 125 | 1912 | 1949 | 38 | 3 | 28 |
| Ren | 124 | 1991 | 2016 | 26 | 5 | 14 |
| Shoemaker | 115 | 1916 | 1964 | 49 | 2 | 0 |
| Holsinger | 114 | 1966 | 2016 | 51 | 2 | 4 |
| Bazikalova | 111 | 1935 | 1975 | 41 | 3 | 4 |
| Bellan-Santini | 108 | 1965 | 2008 | 44 | 2 | 0 |
| Mayer | 106 | 1882 | 1920 | 39 | 3 | 80 |
| Krapp-Schickel | 101 | 1968 | 2015 | 48 | 2 | 0 |
| Dybowsky | 97 | 1874 | 1874 | 1 | 97 | 3 |
| Hirayama | 95 | 1978 | 1993 | 16 | 6 | 0 |
| Hughes | 95 | 2006 | 2016 | 11 | 9 | 0 |
| S. Karaman | 92 | 1929 | 1960 | 32 | 3 | 10 |
